# Supplementary material for: An Analysis of Sport-Specific Pain Symptoms through Inter-Individual Training Differences in CrossFit
Source: Sports (Basel). 2021 May 19;9(5):68. doi: 10.3390/sports9050068 (PMC8160709; doi:10.3390/sports9050068)
Supplement: Supplementary file 1 [file sports-09-00068-s001.zip › sports-1142858-supplementary.pdf]

sports

Supplementary Materials

An Analysis of Sport-Specific Pain Symptoms through Inter-Individual Training Differences in CrossFit

Maria A. Bernstorff [1,*], Norman Schumann [2], Nader Maai [1], Thomas A. Schildhauer [1] and Matthias Königshausen [1]

[1] Medical Department of Ruhr University of Bochum, University Hospital Bergmannsheil Bochum, Bürkle de la Camp-Platz 1, 44789 Bochum, Germany; maria.bernstorff@gmail.com (M.A.B.)nader.maai@bergmannsheil.de (N.M.); thomas.schildhauer@bergmannsheil.de (T.A.S.); matthias.königshausen@gmail.com (M.K.)

[2] Department of Mathematics, Ruhr-University Bochum, Universitätsstraße 150, 44801 Bochum, Germany; norman.schumann@ruhr-universität-bochum.de

* Correspondence: maria.bernstorff@gmail.com; Tel.: (0049-1522-2926-817)

Citation: Bernstorff, M.A.; Schumann, N.; Maai, N.; Schiildhauer TA, Königshausen, M. An Analysis of Sport-Specific Pain Symptoms through Inter-Individual Training Differences in CrossFit. *Sports* **2021**, *9*, 68. https://doi.org/10.3390/sports9050068

Publisher's Note: MDPI stays neutral with regard to jurisdictional claims in published maps and institutional affiliations.

Copyright: © 2021 by the authors. Licensee MDPI, Basel, Switzerland. This article is an open access article distributed under the terms and conditions of the Creative Commons Attribution (CC BY) license (http://creativecommons.org/licenses/by/4.0/).

Table S1. Translated questionnaire: list of all questions recorded in the online questionnaire plus graphics to identify the individual pain regions.

**Athlete and Training Characteristics**
1) How many years have you been doing CrossFit?
2) How many hours a week do you do CrossFit? (Enter your amount of training in hours / week)
3) Do you take part in competitions? Yes/ No
to 3) If yes: RX/advanced/scaled?
4) Do you train according to a training plan designed for you?
To 4) If so, by whom or how was the questionnaire drawn up? Personal Trainer/Online/Self Employed/Employed/Another Source
To 4) If so, do you still take part in the course program of the box? Yes/No
5) Do you warm up regularly before training? Yes/No
6) Are you doing a cool down? Yes/now and then/never
7) Include some of your 1 RM benchmarks: Backsquat/Frontsquat/Deadlift/Power Snatch/Strict Press/Power Clean
8) Do you master: Strict pull-up/Kipping pull-up/Butterfly pull-up/Ring muscle-up/Bar muscle-up/Pistol squats/Handstand walk/Strict handstand push-up
9) Do you do specific exercises for small muscle groups in addition to CrossFit? Yes/No
To 9) If yes, tick for which muscle groups: rotator cuff/scapula retractors/hamstrings/gluteus medius
10) Did you do any other sport regularly before CrossFit? Yes/No
To 10) If so, which one?
To 10) How many years did you do your main sport?
To 10) Do you still do this sport? Yes/No
To 10) Did you practice this sport as a competitive sport? or do you still do it as a competitive sport? Yes/No
**Sport-Specific Injuries**
11) Have you already had to pause your sport because of an injury you sustained while doing CrossFit? Yes/No
12) Are you currently or have you had pain with individual CrossFit exercises in the past six months? Yes/No
To 12) If so, state in the following question at which point this pain is or was. Please use the illustration below as a guide.
To 12) If yes, tick where this pain is or was:
1—hand, wrist/2—elbow/3—shoulders/4—neck, cervical spine/5—upper back, thoracic spine/6—lower back, lumbar spine/7—hips, hip joint/8—sacrumiliac joint, SIJ/9—knee /10—foot/11—ankle
13) For each number ticked above, give the exercises in which you have or have had the pain / injuries?
14) When you no longer have the pain, how did you get it under control?
Omission of individual exercises/technique training of individual exercises/strength building in order to be able to perform certain exercises better/surgery/physiotherapy
To 14) If an operation was necessary, which one?

Table S2. Categorization of the one-repetition max (female): division of the given weights into five groups for better comparability with regard to the pain locations, N = number of athletes given their RM in this category.

Exercise	Group 1	Group 2	Group 3	Group 4	Group 5
Backsquat	<50kg	50–65 kg	65–80 kg	80–95kg	>95 kg
N	19	29	48	55	39
Frontsquat	<50 kg	50–60 kg	60–70 kg	70–80 kg	>80 kg
N	45	24	40	33	38
Strict Press	<25 kg	25–30 kg	30–35 kg	35–40 kg	>40kg
N	17	21	32	44	62
Power Snatch	<20kg	20–30 kg	30–40 kg	40–50 kg	>50 kg
N	7	18	48	68	36
Power Clean	<35 kg	35–45 kg	45–55 kg	55–65 kg	>65 kg
N	19	22	36	54	53
Deadlift	<60 kg	60–80 kg	80–100 kg	100–120 kg	>120 kg
N	12	29	47	64	41

Table S3. Total number of athletes being capable of mastering certain skills.

Mastered Skill	N (Male)	N (Female)
Strict Pul -up	182	127
Kipping Pull-up	157	126
Butterfly Pull-up	81	36
Ring Muscle–up	75	17
Bar Muscle–up	94	20
Pistol Squat	106	125
Handstand Walk	63	39

Table S4. Number of athletes doing accessory exercises regularly.

Accessory exercise	N (male)	N (female)
Rotator cuff	80	75
Shoulder blade retractors	66	72
Hamstrings	58	52
Gluteus medius	38	43
